# Appropriateness of Cotrimoxazole Prophylactic Therapy Among HIV/AIDS Patients in Public Hospitals in Eastern Ethiopia: A Retrospective Evaluation of Clinical Practice

**DOI:** 10.3389/fphar.2018.00727

**Published:** 2018-07-10

**Authors:** Mekonnen Sisay, Dida Bute, Dumessa Edessa, Getnet Mengistu, Firehiwot Amare, Tigist Gashaw, Temesgen Bihonegn

**Affiliations:** ^1^Department of Pharmacology and Toxicology, School of Pharmacy, College of Health and Medical Sciences, Haramaya University, Harar, Ethiopia; ^2^School of Pharmacy, College of Health and Medical Sciences, Haramaya University, Harar, Ethiopia; ^3^Department of Clinical Pharmacy, School of Pharmacy, College of Health and Medical Sciences, Haramaya University, Harar, Ethiopia; ^4^Department of Animal Health, Kombolcha College of Agriculture, Wollo University, Kombolcha, Ethiopia

**Keywords:** cotrimoxazole, drug use evaluation, prophylaxis, HIV/AIDS, Eastern Ethiopia

## Abstract

**Background:** Cotrimoxazole prophylactic therapy (CPT) is a feasible, cost-effective, and safe way of using cotrimoxazole intervention to reduce HIV/AIDS related morbidities and mortalities associated with opportunistic infections. Despite its effectiveness in reducing the incidence of opportunistic infections, the actual drug utilization process has been shown to deviate from World Health Organization (WHO) guideline in Ethiopia. This study, therefore, aims to evaluate CPT among HIV/AIDS patients in Jugel Hospital (JH), Harar and Dilchora Referral Hospital (DRH), Dire Dawa, Eastern Ethiopia.

**Methods:** A cross sectional study was conducted to evaluate the use of cotrimoxazole as prophylactic therapy. In this study, 556 medical records (305 in JH and 251 in DRH) of HIV/AIDS patients who had been taking CPT within September 2015–August 2016 were reviewed. Systematic random sampling was employed to obtain medical records from the sampling frame. Data were abstracted from the patient medical records using structured checklist customized from the WHO guideline. The data were entered into Epi-data 3.1 and exported to and analyzed with statistical Package for Social Sciences (SPSS) version 20. The finding was evaluated against the WHO guideline on the use of cotrimoxazole prophylaxis in HIV/AIDS patients. Descriptive statistics was used to present the data in tables, figures and pie chart.

**Results:** Majority of the HIV/AIDS patients who had been taking CPT were adults (95.9%), female (61.2%), married (43.7%), Orthodox Christian (54.3%), and attended primary school (40.1%). At the initiation of CPT, most of the patients were at WHO clinical stage III (40.8%). The major comorbid illnesses identified were tuberculosis and pneumocystis-jiroveci pneumonia. Initially, majority of the patients were at CD_4_ count of less than 350 cells/mm^3^ (n = 504, 90.6%). Greater proportion of patients started CPT prior to initiating antiretroviral therapy (ART). Most of the patients took CPT for greater than 6 months. The primary reasons for premature discontinuation of CPT were CD4 greater than 350 cells/mm^3^, severe sulfa allergy and first trimester of pregnancy. Generally, the use of cotrimoxazole prophylaxis was consistent with the WHO guideline for indication to start (n = 519, 93.3%) and dose (n = 552, 99.28%), despite the presence of contraindication in 6.65% patients.

**Conclusion:** In reference to the WHO guideline, the use of CPT was found to be fully appropriate in nearly two-thirds of HIV/AIDS patients. For the rest patients, inappropriate use of cotrimoxazole was observed based on the WHO criteria for initiation, discontinuation, continuation and dose with rate of discontinuation being the dominant one. Such practice may lead to adverse health outcomes including adverse drug reactions and negative treatment outcome.

## Introduction

Drug use evaluation (DUE) is a means by which performance is improved upon focusing on overall drug utilization processes to achieve better therapeutic outcomes (WHO, 2007). DUE helps identify drug-related problems at various transition points of drug therapy (Diriba et al., 2008; Leung et al., 2011). Specifically, antimicrobials have clinically profound roles in battle against infectious diseases despite the fact that the ability of these agents to cure or prevent infections is not infinite. At present, antimicrobial resistance, attributable to inappropriate use of antimicrobials, threatens the effectiveness of available agents toward infectious diseases (Leung et al., 2011; Defersha et al., 2014; Gebresillassie et al., 2016).

Cotrimoxazole is a broad spectrum antimicrobial agent targeting a wide range of aerobic gram positive and gram negative bacteria, fungi and protozoa (Tsadik et al., 2015). WHO reports that cotrimoxazole prophylactic therapy (CPT) is a feasible, well tolerated and economically viable intervention for HIV/AIDS patients to reduce the incidence of opportunistic infections (WHO, 2014). Cotrimoxazole is preferable for both primary and secondary prophylaxis of pneumocystis-jiroveci pneumonia (PJP) in adults and adolescents (Geresu et al., 2014). The recommended dose of cotrimoxazole for adults is double strength tablet (960 mg daily) or two single strength 480 mg tablets daily. The dosing of CPT for children (<14 years) is optimized based on their age or body weight bands (WHO, 2014; Gebresillassie et al., 2016).

Wide spread utilization of cotrimoxazole prophylaxis may contribute for emergence of antimicrobial resistance. It is, therefore, prudent to fine-tune the use of cotrimoxazole as prophylactic therapy only to those patients who will be benefited from it. Accordingly, the WHO and Joint United Nation program on HIV/AIDS (UNAIDS) have recommended CPT for HIV/AIDS patients in Africa with symptomatic HIV diseases (WHO clinical stage 3 or 4) and individuals who have a CD4 count of less than or equal to 350 cells/mm^3^ (WHO, 2014). PJP is the most common AIDS-defining opportunistic infection and CPT was shown to effectively prevent PJP in patients with clinical evidence of immune suppression (Diriba et al., 2008; Bardfield et al., 2014; Mekonnin and Gashe, 2015).

A study conducted by Alemayehu et al. (2017) revealed that the overall prevalence of opportunistic infections among HIV/AIDS patients taking ART was 45.3 and 6.9% of them had more than one infections in Wolaita zone, Southern Ethiopia. By the same token, the overall prevalence of opportunistic infections including PJP among HIV/AIDS patients on ART was 48% in Eastern Ethiopia (Mitiku et al., 2015). Despite clinically sound effectiveness in reducing the incidence of opportunistic infections (Tariku et al., 2015), there are some evidences describing about the inappropriate utilization of cotrimoxazole prophylaxis in different areas of Ethiopia (Diriba et al., 2008; Geresu et al., 2014; Mekonnin and Gashe, 2015; Gebresillassie et al., 2016). Therefore, this study aims to evaluate CPT among HIV/AIDS patients in Eastern segments of the country.

## Materials and Methods

### Study Area, Design and Period

The study was conducted in two public hospitals namely: JH, Harari regional state, Harar and Dilchora Referral Hospital (DRH), Dire Dawa city administration, Eastern Ethiopia. JH has three main ward including medical, surgical and gynecology and obstetrics wards. Besides, it provides service for medical, gynecology, eye, dental and pediatric outpatient departments. It also has HIV and TB clinics. The second study area, DRH is located at Dire Dawa 515 km away from Addis Ababa, 311 km from Djibouti and 52 km from Harar town. DRH is a tertiary care hospital having different wards including medical, gynecology and obstetrics, pediatrics and surgical ward. It delivers diversified health services and clinics including the emergency services, maternal and child health, dental clinic, psychiatry clinic, laboratory, X-ray and HIV clinics. A cross sectional study design was employed to evaluate medical records of HIV/AIDS patients who were on CPT at JH and DRH within Sep 2015-Aug 2016. The study was conducted from March to June, 2017.

### Population

Medical records of HIV/AIDS patients who had been on CPT in the HIV clinics of JH and DRH, within September 2015 to August 2016 were considered for inclusion in the study. However, patients with incomplete and unclear medical records were excluded.

### Sample Size Determination and Sampling Techniques

For each setting, the sample size was calculated according to single population proportion formula (Z_α_^2^pq/d^2^). The proportion of CPT use among HIV/AIDS patients (P) was taken from previous study for both settings (*P* = 32%) (Gebresillassie et al., 2016). With 95% confidence level (z) and 5% margin of error (d), as well as the size of study populations [those patients who had been on CPT during the review period (1245 and 724 for JH and DRH, respectively)] were considered for sample size determination. The final sample sizes calculated and adjusted were found to be 305 and 251 for JH and DRH, respectively. Sampling units were selected using systematic random sampling from each study population in respective setting after complete list (sampling frame) of medical records had been prepared in chronological order. The sampling intervals into which samples were drawn were based on the aforementioned study population and calculated sample size. For JH, K = N_JH_/n = 1245/305 = 4; where K is the sampling interval; N is the size of the study population in JH and n is the final sample size. Once the starting number from the first four medical records had been randomly selected (number 2), samples were drawn in every kth (4th) medical records until the calculated sample size was reached. The same procedure was followed for calculating sampling interval in DRH: K = N_DRH_/n = 724/251 = 3. In this setting, samples were taken every third once the starting point (number 1) was selected by simple random sampling from the first three medical records of patients on CPT.

### Data Collection Process

Every relevant information was abstracted from the sampled medical records of patients who were on CPT within the window of review period and recorded into data collection checklist designed and customized from the WHO for DUE of cotrimoxazole as prophylaxis (WHO, 2014). Prior to starting data collection, training was given to four data collectors (one nurse and one druggist in each setting) on how to handle the overall data collection process and appropriately collect essential data for decision making as per the WHO guideline.

### Data Quality Control

Pretest was conducted in Hiwot Fana Specialized University Hospital on 50 medical records of HIV/AIDS patients who were on CPT to assure that the data collection format is feasible in closely related setting. Necessary adjustment was made accordingly on the checklist considering temporal and spatial differences. Data cleaning was also performed on daily basis.

### Data Processing, Analysis and Evaluation

Data were entered in to Epi-data version 3.1 and exported and analyzed by using Statistical Package for Social Sciences (SPSS) version 20 (IBM statistics, Armonk, NY, United States). Descriptive statistics (frequency and percent) was determined to summarize sociodemographic and baseline clinical characteristics, timing of CPT initiation and duration of HIV/AIDS patients on CPT prior to discontinuation. However, critical evaluation was conducted as per the WHO guideline for the use of cotrimoxazole as prophylaxis despite the presence of contraindication, suggested reasons for premature discontinuation, and overall appropriateness of cotrimoxazole use as prophylaxis in these individuals considering the dose, initiation criteria, continuation and discontinuation in both settings (WHO, 2014).

## Results

### Sociodemographic Characteristics

In this study, a total of 556 medical records of HIV/AIDS patients who had been on CPT were reviewed for evaluation of cotrimoxazole use as prophylactic therapy as per the WHO guideline. Concerning the sociodemographic characteristics, the majority of patients were adolescents and adults (*n* = 533, 95.9%), female (*n* = 340; 61.2%), married (*n* = 243, 43.7%), Orthodox Christian (*n* = 302, 54.3%), and attended primary school (*n* = 223, 40.1%). From those female who were pregnant, 6(31.6%) patients were at first trimester meanwhile using cotrimoxazole in the review period (**Table 1**).

**Table 1:** Sociodemographic and obstetric characteristics of HIV/AIDS patients who were on cotrimoxazole prophylactic therapy (CPT) in selected public hospitals (JH and DRH), Eastern Ethiopia within September 2015–August 2016.

Variables		JH (%)	DRH (%)	Overall (%)
Age (y)	<5	0 (0.0)	10 (4.0)	10 (1.8)
	5–14	7 (2.3)	6 (2.4)	13 (2.3)
	>14	298 (97.7)	235 (93.6)	533 (95.9)
Sex	Male	101 (33.1)	115 (45.8)	216 (38.8)
	Female	204 (69.9)	136 (54.2)	340 (61.2)
Marital status	Married	148 (48.2)	95 (37.8)	243 (43.7)
	Single	40 (13.1)	54 (21.5)	94 (16.9)
	Widowed	45 (14.8)	51 (20.3)	96 (17.3)
	Divorced	65 (21.6)	35 (13.9)	100 (17.9)
Religion	Muslim	77 (25.2)	92 (36.7)	169 (30.4)
	Orthodox	193 (63.3)	109 (43.4)	302 (54.3)
	Protestant	33 (10.8)	46 (18.3)	79 (14.2)
	Others^∗^	2 (0.7)	4 (1.6)	6 (1.1)
Educational level	No formal education/under age	72 (23.6)	50 (19.9)	122 (21.9)
	Primary school	129 (42.3)	94 (37.5)	223 (40.1)
	Secondary school	72 (23.6)	74 (29.5)	146 (26.3)
	College and above	30 (10.5)	33 (13.1)	63 (11.3)
Pregnancy status	Yes	17 (8.34)	2 (1.47)	19 (5.6)
	1st trimester	4 (23.53)	2 (100)	6 (31.6)
	2nd trimester	7 (41.17)	0 (0.0)	7 (36.8)
	3rd trimester	6 (35.3)	0 (0.0)	6 (31.6)
	No	187 (91.66)	134 (98.53)	321 (94.4)

*JH, Jugel hospital; DRH, Dilchora referral hospital; y, year; CPT, cotrimoxazole prophylactic therapy. Others^∗^ refer to Catholic, Pagan, or Jova witness believers*.

### Baseline Clinical Characteristics

Concerning the baseline clinical characteristics, most of the patients (*n* = 227, 40.8%) were at WHO clinical stage III. TB and PJP were found to be the most prevalent co-infections in this study. The overall prevalence of HIV-TB co-infection at the initiation of ART was found to be 20.3% (*n* = 113). The baseline laboratory findings revealed that majority (*n* = 504, 90.6%) of the patients had a CD4 count less than 350 cell/mm^3^. Few patients also had diagnosis of severe renal insufficiency (Scr > 1.5 mg/dl), and severe pancytopenia (anemia, neutropenia and thrombocytopenia) in both healthcare settings (**Table 2**).

**Table 2:** Baseline clinical characteristics of HIV/AIDS patients on ART follow up clinic of JH and DRH, Eastern Ethiopia, September 2015–August 2016.

Baseline characteristics		JH (%)	DRH (%)	Overall (%)
WHO clinical stage	I	122 (40.0)	26 (10.4)	148 (26.6)
	II	41 (13.4)	73 (29.1)	114 (20.5)
	III	113 (37.04)	114 (45.4)	227 (40.8)
	IV	29 (9.6)	38 (15.1)	67 (12.1)
Comorbid illnesses	TB	67 (22.0)	46 (18.3)	113 (20.3)
	PJP	10 (3.3)	54 (21.5)	64 (11.5)
	Oral ulcer	25 (8.2)	28 (11.2)	53 (9.5)
	Toxoplasmosis	9 (3.0)	6 (2.4)	15 (2.7)
	Extra-pulmonary TB	15 (4.9)	8 (3.2)	23 (4.1)
	Herpes	10 (3.3)	5 (2.0)	15 (2.7)
	Others^∗^	39 (12.8)	59 (23.5)	98 (17.6)
	No comorbidity	122 (40.0)	28 (11.2)	150 (26.9)
	No documentation^∗∗^	8 (2.6)	17 (6.8)	25 (4.5)
Laboratory results	CD4 < 350 cells/mm^3^	274 (89.8)	230 (91.6)	504 (90.6)
	Hgb < 7 g/dl	6 (1.96)	5 (2.0)	11 (1.98)
	Platelet count < 50,000 cell/ml	2 (0.66)	2 (0.8)	4 (0.7)
	Neutrophil <750 cells/mm^3^	6 (1.96)	4 (1.6)	10 (1.8)
	Scr > 1.5 mg/dl	6 (1.96)	2 (0.8)	8 (1.4)
	ALT in Female (>90 IU/L) or male (>115 IU/L)	0 (0.0)	0 (0.0)	0 (0.0)

^∗^*Others = fungal infections, chronic diarrhea. TB, tuberculosis; PJP, pnemocystic-jiroveci pneumonia, JH, Jugel Hospital; DRH, Dilchora Referral Hospital. ^∗∗^Refers the presence of undocumented comorbidity expected from additional drugs taken*.

### Highly Active Antiretroviral Therapy (HAART) Regimens

**FIGURE 1:**
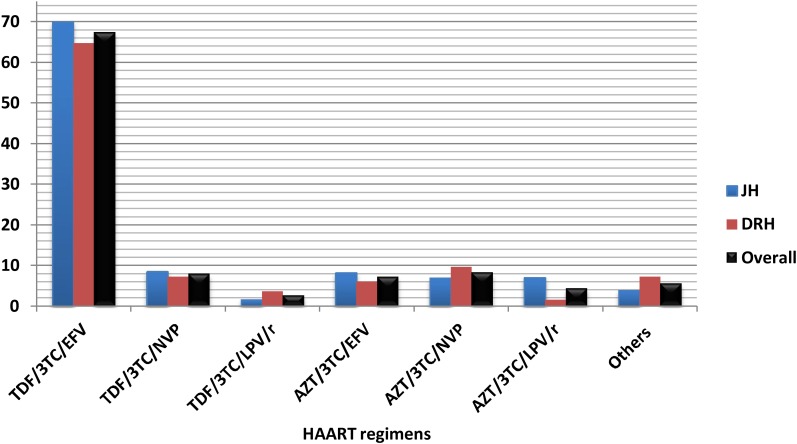
Highly active antiretroviral therapy (HAART) regimens taken by HIV/AIDS patients who were on cotrimoxazole prophylactic therapy (CPT) at JH and DRH, from September 2015 to August 2016. (Others: AZT/3TC/ATZ/r, TDF/3TC/ATZ/r, ABC/3TC/LPV/r.) TDF, Tenofovir; 3TC, lamivudine; EFV, efavirenz; NVP, Nevirapine; LPV/r, lopinavir with ritonavir; AZT, zidovudine; ATZ/r, Atazanavir with ritonavir; ABC, Abacavir. HAART, Highly active antiretroviral therapy.

The majority (67.4%) of HIV/AIDS patients were taking tenofovir (TDF)-based regimens with TDF/3TC/EFV being the most prevalent regimen in these hospitals (**Figure 1**).

### Utilization Pattern of Cotrimoxazole

In reference to consolidated WHO guideline, 552 (99.28%) patients received correct dose of cotrimoxazole. The majority of patients initiated CPT prior to ART (*n* = 268, 48.2%) (**Table 3**). With regard to length of stay (in month) of patients who were on CPT during the review period, greater proportion of the patients (*n* = 214, 38.5%) took CPT for greater than 6 months (**Table 3**). From all patients, 239 (43.0%) patients discontinued it due to various reasons with immunological recovery (CD4 count greater than 350 cells/mm^3^) being the primary reason (*n* = 115, 48.1%). Other documented reasons for cessation of CPT were severe sulfa-related allergic reactions and first trimester of pregnancy (**Table 3**). Cotrimoxazole was used in 37 (6.65%) cases despite the presence of contraindications. As per the WHO guideline, the specific contraindications were sulfa allergy, renal insufficiency at baseline (serum creatinine > 1.5 mg/dl), first trimester of pregnancy, severe anemia (Hgb < 7 g/dl), severe neutropenia (neutrophil < 750 cells/mm^3^) and severe thrombocytopenia (platelet < 50,000 cells/ mm^3^) (**Table 3**).

**Table 3:** Timing of CPT initiation, duration of therapy, reasons for discontinuation and contra-use among HIV/AIDS patients at JH and DRH, from September 2015 to August 2016.

Indicator variables		JH (%)	DRH (%)	Overall (%)
Timing of CPT initiation	CPT before ART (Weeks)	168 (55.1)	100 (39.8)	268 (48.2)
	<2	58 (34.5)	27 (27.0)	85 (31.7)
	2	82 (48.8)	12 (12.0)	94 (35.1)
	>2	28 (16.7)	61 (61.0)	89 (33.2)
	CPT and ART simultaneously	125 (41.0)	78 (31.1)	203 (36.5)
	CPT after ART (weeks)	12 (3.9)	73 (29.1)	85 (15.3)
	<2	1 (8.3)	9 (12.3)	10 (11.8)
	2	4 (33.3)	2 (2.7)	6 (7.1)
	>2	7 (58.3)	62 (84.9)	69 (81.2)
Duration on CPT (Months)	<1	38 (12.5)	36 (14.3)	74 (13.3)
	1–3	103 (33.8)	87 (34.7)	190 (34.2)
	3–6	55 (18.0)	23 (9.2)	78 (14.0)
	>6	109 (35.7)	105 (41.8)	214 (38.5)
Reasons for discontinuation	CD4 > 350 cells/mm^3^	71 (49.3)	44 (46.3)	115 (48.1)
	Severe allergic reactions	14 (9.7)	12 (12.6)	26 (10.9)
	First trimester of pregnancy	2 (1.4)	1 (1.1)	3 (1.3)
	Not documented	57 (39.6)	38 (40.0)	95 (39.7)
	Subtotal	144 (47.2)	95 (37.8)	239 (43.0)
Contra-use	Sulfa allergy	2 (9.1)	2 (13.3)	4 (10.8)
	Renal insufficiency	6 (27.3)	1 (6.7)	7 (18.9)
	First trimester of pregnancy	2 (9.1)	1 (6.7)	3 (8.1)
	Severe anemia	6 (27.3)	5 (33.3)	11 (29.7)
	Severe neutropenia	4 (18.2)	4 (26.7)	8 (21.6)
	Sever thrombocytopenia	1 (4.5)	2 (13.3)	3 (8.1)
	Subtotal	22 (7.21)	15 (5.97)	37 (6.65)

*JH, Jugel Hospital; DRH, Dilchora Referral Hospital; CPT, cotrimoxazole prophylactic therapy; ART, antiretroviral therapy*.

### Appropriateness of CPT

**FIGURE 2:**
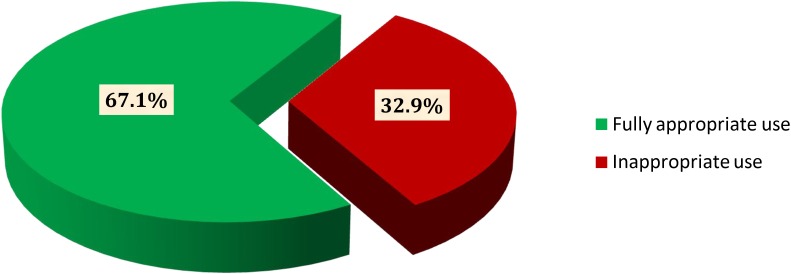
Overall evaluation of CPT among HIV/AIDS patients at JH and DRH from September 2015 to August 2016.

**FIGURE 3:**
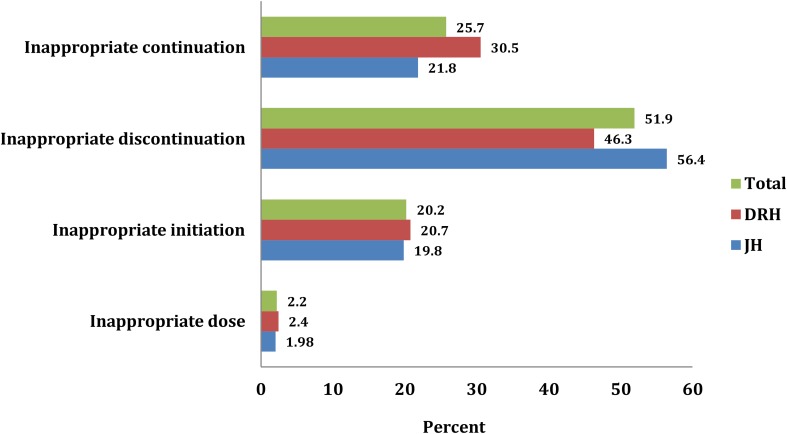
Inappropriate CPT with regard to initiation, continuation, discontinuation and dosage among HIV/AIDS patients at JH and DRH from September 2015 to August 2016.

Considering all possible clinical and sociodemographic aspects, 373 (67.1%) patients were found to be fully appropriate (**Figure 2**). The remaining patients, 183 (32.9%) used it inappropriately including inappropriate initiation without considering WHO guideline (20.2%) incorrect dosage consideration (2.2%), inappropriate continuation of CPT in spite of the presence of contraindication and/or beyond the optimum duration of therapy (25.7%), and inappropriate discontinuation of therapy (51.9%) (**Figure 3**).

## Discussion

This study tried to evaluate the appropriateness of cotrimoxazole use among HIV/AIDS patients in Ethiopia. Such evaluation is used as a performance improvement method and a basis for designing and implementing interventional strategies once the major problem areas are identified (WHO, 2007). Antimicrobial agents are given greater emphasis by WHO since these agents are highly utilized but commonly misused class of drugs. The misuse of antimicrobials is strongly linked as a major drive for the emergence of antimicrobial resistance (WHO, 1993). Cotrimoxazole is a commonly utilized broad spectrum antimicrobial agent for the treatment of infections caused by bacteria, fungi and protozoa. WHO recommends the use of cotrimoxazole as CPT in HIV/AIDS patients provided all the criteria are fulfilled (WHO, 2014). Therefore, evaluating the use of cotrimoxazole as prophylactic therapy among patients helps understand the way that cotrimoxazole has been utilized in these hospitals.

In this study, the majority of patients, who had been taking CPT, were adults, female, orthodox Christian, married, and attended primary school in both hospital settings. This data is concordant with HUTRH and JUSH where female patients accounted for 60.7 and 70.9%, respectively (Deresse and Alemayehu, 2009; Defersha et al., 2014). The same is true for other studies conducted in different parts of Ethiopia where the greater proportion of patients involved in the study were females and were with age greater than 14 years (Diriba et al., 2008; Mekonnin and Gashe, 2015; Gebresillassie et al., 2016). This might be due to the fact that females are more likely to be infected with HIV due to physiologic differences (recipients are more risky than donors).

Coming to the baseline clinical characteristics and criteria for initiation of CPT, majority of patients (40.8%) presented with WHO clinical stage III. From patients who manifested comorbid illnesses, the majority of them developed TB and PJP as AIDS-indicator opportunistic infections in advanced stage of HIV during the initiation of CPT. Most of the patients attended the hospital settings with CD_4_ cell count less than 350 cells/mm^3^. This study is in line with previous studies conducted in different parts of Ethiopia where the majority of patients were categorized at WHO clinical stage III during initiation of CPT. These studies also reported that majority of the patients presented with CD4 cell count less than or equal to 350 cell/mm^3^ (Geresu et al., 2014; Mekonnin and Gashe, 2015; Gebresillassie et al., 2016).

Regarding the initiation criteria, a total of 37 patients initiated CPT inappropriately at early stage of HIV (WHO clinical stage I or stage II) without noticeable comorbid illnesses plus CD4 count greater than 350 cell/mm^3^ and/or despite the presence of hematological and clinical chemistry test results that contradicted the use of CPT for some patients. The majority (*n* = 519, 93.3%) of patients initiated CPT in line with WHO guideline for indication (WHO, 2014). This finding is further supported by the studies conducted by Geresu et al. (2014) at BMH, Mekonnin and Gashe (2015) at Jimma Health Center, and Gebresillassie et al. (2016) at Gondar University Specialized Hospital (GUSH) (Gebresillassie et al., 2016) where the use of CPT among HIV/AIDS patients was consistent with the WHO guideline for indications at the level of 97.98, 96.83, and 75.75%, respectively.

Cotrimoxazole prophylactic therapy is a recommended prophylaxis of opportunistic infections in patients with severe or advanced HIV clinical disease (WHO stage 3 or 4) and/or for a CD4 count ≤ 350 cells/mm^3^ (WHO, 2014). In settings where severe bacterial infections and/or malaria are highly prevalent, CPT should be initiated regardless of CD4 cell count or WHO clinical stage. Besides, priority must be given for children who are less than 5 years to initiate CPT regardless of CD4 count and/or WHO clinical stage (WHO, 2013, 2014). Cotrimoxazole was found to be associated with a 37% reduction in mortality and the reduction is dependent on the duration of CPT and baseline CD4 count (Yazdanpanah et al., 2005; Cheng et al., 2015). CPT is a cost-effective intervention with expected high impact on morbidity and mortality reduction in HIV-infected adults in resource-limited settings. According to Hassani et al. (2015), benefits are seen in both pre-ART and ART taking populations. Hasse et al. (2014) reported that CPT may reduce the risk of TB in ART-naive persons and, there was also a trend of a protective effect even among patients on ART. Therefore, routine CPT should be administered to all HIV-infected people with active-TB disease regardless of CD4 cell count (WHO, 2014).

In both hospitals, larger proportion of patients initiated CPT prior to ART followed by those who started CPT and ART concurrently. The same is true for the study conducted by Gebresillassie et al. (2016) at GURH where the percentage of patients who initiated CPT before ART as well as CPT and ART at the same time were 52.7 and 45.1%, respectively. Among patients who were on CPT, four of them took inappropriate (under) dose compared to WHO guideline with reference to age category. Generally, 99.28% patients utilized CPT in line with the WHO guideline for dose (WHO, 2014). Similarly, previous study indicated that among patients who took inappropriate dose (3.59%), most of them (2.79%) were subjected to under dose (Mekonnin and Gashe, 2015). Besides, the use of CPT was consistent with the guidelines in the rationale for dose in 99.62, 85.9, and 96.77% patients at GURH, JURH and BMH, respectively (Defersha et al., 2014; Geresu et al., 2014; Gebresillassie et al., 2016). It is scientifically justifiable to start CPT prior to ART in majority of patients. In advanced HIV/AIDS (stage III or IV) and/or in cases of severe reduction in CD4 count, nevirapine based ART regimen may be initiated for better immune recovery. However, this therapy is commonly associated with allergic reactions and hepatotoxicity. Therefore, to distinguish the hypersensitivity reaction caused by cotrimoxazole from nevirapine, CPT should be started in advance and the ART can be initiated two or more weeks later. In certain situations, however, CPT and ART can be initiated concomitantly. This can occur when there is non-nevirapine based ART regimens and when the potential benefit of starting both therapies outweighs any potential risk. Studies have reported that cotrimoxazole provides a continuous protective effect even after ART initiation. Yet, the implementation of CPT remains a challenge in low- and middle-income countries (Gupta et al., 2014; WHO, 2014).

The larger proportion of patients continued CPT for more than 6 months in both settings. The WHO guideline stressed that patients who started CPT should continue for at least 6 months (WHO, 2014). Gebresillassie et al. (2016) also reported that the majority (73.9%) of patients took cotrimoxazole for more than 6 months.

Considering the ART regimens, most of the patients were on TDF-based regimens (TDF/3TC/EFV or TDF/3TC/ NVP) which is currently the preferred first line regimen in Ethiopia. TDF based regimen has also been considered to be the safest regimen associated with low degree of metabolic toxicity (FMHACA, 2014). Moreover, Deeks and Perry stressed that adherence to treatment was maintained or improved with the TDF/3TC/EFV single-tablet regimen over their previous more complex regimen finding it easier to follow (Deeks and Perry, 2010; Doherty et al., 2013; WHO, 2016). There has been a negligible drug interaction of CPT with this regimen.

In this study, the primary reasons for discontinuation of CPT were found to be CD4 count greater than 350 cell/mm^3^, severe sulfa drug allergy and first trimester of pregnancy. CPT can be discontinued in adults (including pregnant women) with HIV infection who are clinically stable on ART, with evidence of immune recovery and viral suppression (WHO, 2013, 2014). To this end, Bwakura-Dangarembizi et al. (2014) indicated that participants who stopped cotrimoxazole had higher rates of hospitalization or death than those who continued it. Prophylaxis with cotrimoxazole is a recommended intervention of proven benefit that could serve not only as an initial step toward improving pediatric care in young children with limited access to ART, but also as an important complement to ART in resource-limited settings (Zachariah et al., 2007; Bardfield et al., 2014). The magnitude of discontinuation is higher than the study conducted by Geresu et al. (2014) (20.97%) but lower than that of Diriba et al. (2008) (76.6%). Furthermore, Mekonnin and Gashe reported that 70 (27.88%) patients discontinued CPT and the main reason stated for discontinuation was CD4 count greater than 350 cells/mm^3^ (57.33%). However, nine of these patients discontinued the therapy inappropriately (Mekonnin and Gashe, 2015).

This study also revealed that there was substantial contra-use of cotrimoxazole in both settings. These include severe sulfa allergy (rash), severe renal insufficiency, severe pancytopenia (anemia, neutropenia and thrombocytopenia) and first trimester of pregnancy. The current WHO guideline emphasized that, in the presence of such contraindications, CPT should be terminated since the potential risk may outweigh any benefits (WHO, 2014). Gebresilassie et al. (2016) reported that CPT was used in 24 (9.90%) patients despite the presence of contraindications including allergic reactions, severe hepatic disease, severe renal problem and severe pancytopenia, among others.

### Strength and Limitation of the Study

This study tried to evaluate the use of cotrimoxazole as prophylaxis among patients based on standardized criteria customized from current WHO guideline. It is also a clinically sound research that addressed major aspects of the commonly used antimicrobial for HIV infected people in resource limited settings. This study may pave a way forward in indicating the major problem areas for designing interventional strategies. However, the study was not without potential limitations. As it is a retrospective study which actually saves time and resources, complete patient information/records were not available and/or the actual patients could not be addressed for informed decision making unlike the prospective researches. As it is also a cross-sectional study, it could not address the future impact of patients completing or discontinuing the therapy.

## Conclusion

In reference to the WHO guideline, the use of CPT was found to be fully appropriate in almost two-thirds of HIV/AIDS patients. For the remaining patients, inappropriate use of cotrimoxazole was observed based on the WHO criteria for initiation, discontinuation, continuation and dose with rate of discontinuation being the dominant one. Such practice may lead to adverse health outcomes including adverse drug reactions and negative treatment effects. Considering the major problem areas in CPT, a multitude of strategies should be designed to improve the current utilization pattern to adhere to WHO guideline at best.

## Availability of Data and Materials

All the data used for the study is contained within the manuscript.

## Ethics Statement

Ethical approval was sought and received from Institutional Health Research Ethics Review Committee of College of Health and Medical Sciences. A clearance letter was obtained from the College of Health and Medical Sciences, Haramaya University with reference number C/AC/R/D/01/213/17 for conducting this research in both hospitals. Official permission was also requested and received from hospital administrators to start the study in HIV clinic of each hospital. Informed consent was not sought since the study did not directly involve HIV/AIDS patients who were on CPT; however, confidentiality of the patient information was maintained in such a way that the data collection checklist was kept anonymous.

## Author Contributions

MS and DB conceived the study and drafted the proposal. DE, GM, FA, TG, and TB had substantial contribution in the study design and development of data collection checklist. All authors were involved in data acquisition, analysis, interpretation and write up. MS and DE drafted the manuscript. MS also prepared the final draft for publication. All authors read and approved the final version of the manuscript.

## Conflict of Interest Statement

The authors declare that the research was conducted in the absence of any commercial or financial relationships that could be construed as a potential conflict of interest.

## Abbreviations

ART: antiretroviral therapy
BMH: Boru Meda Hospital
CPT: cotrimoxazole prophylactic therapy
DRH: Dilchora Referral Hospital
GURH: Gondar University Referral Hospital
HUTRH: Hawassa University Teaching and Referral Hospital
JH: Jugel Hospital
JUSH: Jimma Specialized Hospital
PJP: pneumocystic-jiveroci pneumonia
HIV/AIDS: Human immunodeficiency virus/Acquired Immune Deficiency Syndrome
WHO: World Health Organization.
